# HPV Genotype Landscape in Anal Squamous Cell Carcinoma Across Primary, Recurrent, and Metastatic Tumor Specimens

**DOI:** 10.3390/cancers18152396

**Published:** 2026-07-25

**Authors:** Rose Seiberth, Hans Michael Kvasnicka, Tina Senff, David Brodmann, Florian Gebauer, Lars Bönicke, Daniel Gödde

**Affiliations:** 1 Department of General, Visceral and Oncological Surgery, Helios University Hospital Wuppertal, University of Witten/Herdecke, Heusnerstraße 40, 42283 Wuppertal, Germany; 2 Institute of Pathology and Molecular Pathology, Helios University Hospital Wuppertal, University of Witten/Herdecke, 42283 Wuppertal, Germany

**Keywords:** anal squamous cell carcinoma, HPV genotyping, human papillomavirus, multiplex PCR, co-infection, type diversity, tumor recurrence, metastasis, Anyplex II HPV28

## Abstract

Background/Objectives: Anal squamous cell carcinoma (SCCA) is an HPV-driven malignancy with rising incidence, yet systematic HPV genotyping across recurrences and metastases is lacking. We characterized the complete HPV genotype distribution across the disease course. Methods: We retrospectively analyzed 213 tumor specimens from 136 patients (82 female, 54 male) with histologically confirmed SCCA, spanning primary, recurrent, and metastatic disease (2009–2019 and from 2020 onwards). Patients contributed one or more specimens depending on the number of sampled disease events; specimens comprised 138 primary biopsies, 55 recurrence biopsies, 2 re-recurrence biopsies, and 18 distant metastases. HPV genotyping was conducted using a validated 28-type multiplex real-time PCR assay (Anyplex™ II HPV28, Seegene Inc.). Results: HPV DNA was detected in 179/213 specimens (84.0%; 95% CI 78.9–88.7) and 125/136 patients (91.9%; 95% CI 86.8–96.3). Across 269 HPV type detections, 24 distinct types were identified, with HPV 16 predominating both per-specimen (90.5%) and per-detection (60.2%) values. Non-HPV-16/18 high-risk types accounted for one in four high-risk detections (54/219; 24.7%; 95% CI 19.4–30.8). HPV positivity differed significantly by specimen type: 90.6% in primary biopsies versus 70.9% in recurrences (*p* = 0.001) and 72.2% in metastases (*p* = 0.038). Co-infections occurred in 24.6% of HPV-positive specimens (95% CI 18.5–31.6), with up to eight types. In metastases, the spectrum narrowed to 4 of 24 types; two harbored HPV 33 as the sole high-risk type. Conclusions: SCCA harbors a substantially broader HPV type spectrum than previously recognized. One in four high-risk detections lies beyond HPV 16/18, and high-risk types other than HPV 16 were the sole high-risk genotype detected in individual metastatic specimens. HPV positivity rates are significantly lower across specimen types, while the metastatic type spectrum converges on a few high-risk genotypes. Broad-spectrum HPV genotyping beyond types 16 and 18 warrants adoption as standard practice in SCCA diagnostics and surveillance, and potential stratification for HPV-directed therapies.

## Simple Summary

Anal squamous cell carcinoma (SCCA) is a rare but increasing cancer predominantly driven by human papillomavirus (HPV) infection. Most studies have focused on HPV type 16 and pre-therapeutic biopsies alone, leaving the full spectrum of HPV types across primary tumors, recurrences, and metastatic specimens largely uncharacterized. Using a validated 28-type multiplex assay, we genotyped 213 tumor specimens from 136 patients, identifying 24 distinct HPV types and a co-infection rate of 24.6%. HPV positivity was significantly lower in recurrent and metastatic specimens than in primary biopsies, non-HPV-16 types were the only high-risk genotype detected in individual metastatic specimens, and over 20% of all detections involved types not covered by current HPV vaccines. As HPV type composition may influence tumor biology and response to immune-based therapies, these findings are relevant not only for prevention and diagnostics but also for the type-informed management of patients with advanced disease.

## 1. Introduction

Squamous cell carcinoma of the anal canal and anal margin (SCCA) is a rare malignancy, accounting for approximately 2–5% of all gastrointestinal cancers. Despite its rarity, incidence rates have been rising steadily in Europe and North America over recent decades, a trend attributed in part to increasing rates of oncogenic human papillomavirus (HPV) infection [1,2]. Among European countries, Germany reported the highest age-standardized anal cancer incidence rates in the period 2008–2012, with 1.65 per 100,000 men and 2.16 per 100,000 women, and upward trends have continued since [3]. The disease predominantly affects individuals in their sixth decade of life, with a peak incidence around the age of 65 years [4].

For most patients with localized SCCA (stages I–III), combined chemoradiotherapy (CRT) according to the Nigro protocol—established in the early 1970s—remains the standard of care [5,6,7]. Surgical salvage through abdominoperineal resection is reserved for patients with CRT failure [6]. In recent years, immune checkpoint inhibitors have emerged as a therapeutic option in advanced SCCA, underscoring the relevance of tumor immunobiology and viral antigen characterization [8,9]. HPV-driven carcinogenesis is closely intertwined with immune dysregulation: chronic inflammation and impaired immune surveillance—for example, in the context of HIV co-infection—facilitate viral persistence, oncogene expression, and immune evasion. While high-risk genotypes drive transformation through the E6/E7 oncoproteins, low-risk genotypes such as HPV 6 and 11 have occasionally been demonstrated within high-grade anal lesions and invasive carcinoma [10], and co-infection with multiple genotypes may modulate the oncogenic process. Therefore, a comprehensive characterization of the full genotype spectrum, including low-risk types and co-infections, is a prerequisite for understanding these interactions.

Human papillomavirus infection is the predominant etiological driver of SCCA, detectable in 70–90% of cases [11,12]. HPV type 16 is the most common oncogenic type and is associated with improved responses to CRT and a favorable prognosis compared with HPV-negative SCCA [13,14]. HPV 18, while less prevalent, represents another established high-risk genotype in this entity [11]. Beyond HPV 16 and 18, more than 14 additional high-risk HPV genotypes have been linked to cervical and other anogenital carcinogenesis; yet, their specific prevalence and pathogenic relevance in SCCA remain incompletely characterized [15,16].

Critically, existing studies on HPV genotyping in SCCA have been predominantly limited to pre-therapeutic primary tumor biopsies, leaving the longitudinal dynamics of HPV infection across the disease course largely unexplored [17,18]. Whether HPV status and type composition are maintained in recurrent or metastatic tumor tissue—and whether type shifts occur during disease progression—has not been systematically investigated. This question is of particular clinical relevance: HPV genotype-specific differences in immunogenicity and response to immune checkpoint blockade have been described in other HPV-associated malignancies, suggesting that comprehensive HPV profiling may carry prognostic and predictive value beyond the binary HPV-positive/negative classification [19,20].

Furthermore, a relevant proportion of SCCA may be attributable to HPV types not covered by current prophylactic vaccines [21,22], though systematic evaluation of this question in large, comprehensively genotyped cohorts remains limited.

To address these gaps, we applied a validated 28-type multiplex PCR assay on 213 tumor specimens from 136 patients with SCCA, encompassing pre-therapeutic primary biopsies, recurrence biopsies, re-recurrence biopsies, and metastatic specimens. We aimed to characterize the complete HPV genotype landscape across all available specimen types, with a focus on type diversity and co-infection patterns.

## 2. Materials and Methods

### 2.1. Study Design and Ethics

This retrospective single-center study was conducted at the Helios University Hospital Wuppertal, University of Witten/Herdecke, Germany. Ethical approval was obtained from the Ethics Committee of the University of Witten/Herdecke (AZ 110/2020, approved on 24 August 2020). All investigations were performed in accordance with the Declaration of Helsinki. This study was supported by Helios Kliniken GmbH (Grant-ID: 2020-0076) and reported in accordance with the STROBE (Strengthening the Reporting of Observational Studies in Epidemiology) guidelines for observational studies (Table S1).

Patient identification was performed through the clinical information system (SAP) using ICD-10 codes C21.0, C21.1, and C44.5, and through the institutional pathology database (DC-Pathos) using full-text search terms including “Analkanal”, “Analrand”, “Analkarzinom”, “Rektumexstirpation”, “HPV”, and “TNM”, restricted to the years 2009–2019 (retrospective arm). A prospective arm was added for patients treated from 2020 onwards. Written informed consent was obtained from all living patients with available contact data; for patients who had been deceased or could not be contacted, a waiver of informed consent was granted under the ethics approval.

### 2.2. Patient and Specimen Selection

*Clinical collective:* Initial database queries identified 145 patients with ICD-10 codes consistent with anal or perianal malignancies. After excluding 3 patients with non-squamous cell carcinomas (adenocarcinomas, vulvar carcinomas, or no evidence of tumor), 142 patients remained. Of these, 6 were excluded due to insufficient or absent tumor material for HPV testing. The final histopathological collective comprised 136 patients (82 female, 54 male).

**Figure 1:**
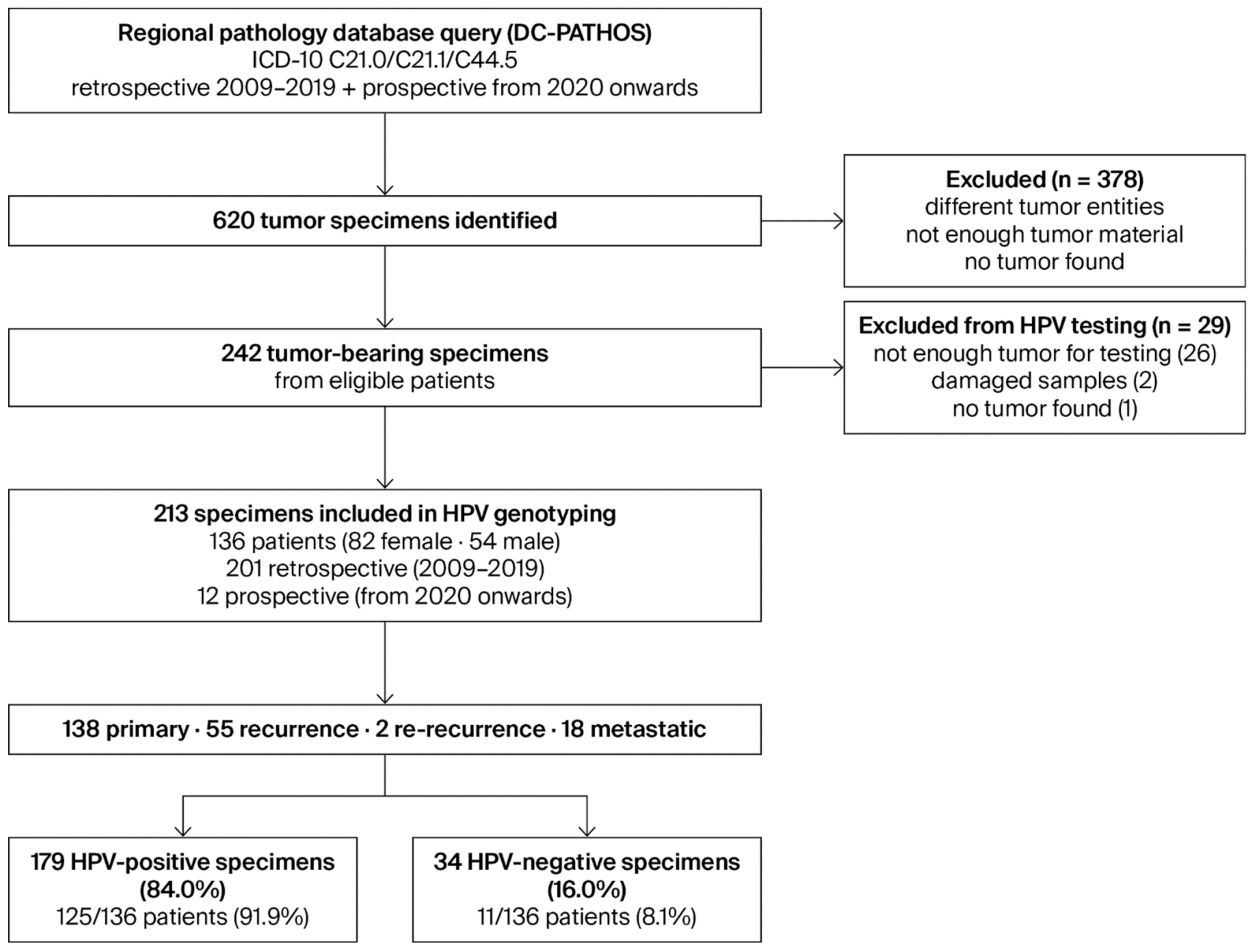
Patient and specimen selection flowchart. Numbers indicate the count of specimens (*n*) at each selection step; boxes on the right give the reasons for exclusion with the corresponding specimen numbers. The final analysis collective comprised 213 specimens from 136 patients.

*Specimen collective:* Pathology database queries (DC-PATHOS; retrospective 2009–2019 and prospective from 2020 onwards) identified 620 tumor specimens. Sequential exclusion of 378 specimens with non-qualifying tumor entities, insufficient tumor material, or no tumor found on review left 242 tumor-bearing specimens from eligible patients; a further 29 specimens were excluded from HPV testing (26 with insufficient tumor for testing, 2 damaged, and 1 with no tumor found), yielding 213 specimens from 136 patients for the final HPV analysis (Figure 1). Of these, 201 derived from the retrospective arm (2009–2019) and 12 from initial prospective cases (from 2020 onwards). Of these, 179 (84.0%) were HPV-positive, and 34 (16.0%) were HPV-negative. Specimen types included 138 pre-therapeutic primary biopsies, 55 recurrence biopsies obtained after prior antitumoral treatment, 2 re-recurrence biopsies, and 18 metastatic specimens. All histopathologically confirmed disease manifestations of a given patient—primary tumor, local recurrence, re-recurrence, and distant metastasis—for which archival FFPE material of sufficient quality was available were included as separate specimens. Therefore, multiple specimens from a single patient corresponded to distinct disease events sampled at different time points across the disease course rather than to repeated sampling of the same lesion.

### 2.3. HPV Genotyping

All tumor specimens were retrieved from the institutional pathology archive as formalin-fixed, paraffin-embedded (FFPE) tissue blocks. Histological evaluation was performed on FFPE tissue blocks to identify and mark tumor-rich areas. Alternatively, FFPE blocks containing exclusively tumor tissue were selected. Genomic DNA was extracted using the Maxwell^®^ 16 FFPE Plus LEV DNA Purification Kit (Promega, Madison, WI, USA) according to the manufacturer’s instructions. Briefly, tissue sections were deparaffinized, digested with proteinase K (supplied with the kit) overnight, and subsequently lysed prior to semi-automated DNA purification on the Maxwell® 16 Instrument (Promega, Madison, WI, USA). DNA concentration was determined using the Quantus™ Fluorometer (Promega, Madison, WI, USA). For PCR analysis, the housekeeping gene β-globin was co-amplified as an internal quality control together with positive and negative controls. No minimum DNA concentration threshold was applied; sample adequacy was defined by successful amplification of a β-globin gene (HBB) fragment. For HPV detection, a sample was considered negative for a given HPV type if no adequate amplification signal was detected after 50 PCR cycles.

HPV genotyping was performed using the Anyplex™ II HPV28 Detection assay (Seegene Inc., Seoul, Republic of Korea; Document No. HP7S-EN190703B-01), a multiplex real-time PCR assay capable of simultaneously detecting and genotyping 28 distinct HPV types: 19 high-risk types (HPV 16, 18, 26, 31, 33, 35, 39, 45, 51, 52, 53, 56, 58, 59, 66, 68, 69, 73, 82) and 9 low-risk types (HPV 6, 11, 40, 42, 43, 44, 54, 61, 70). High- and low-risk designations follow the classification of the International Agency for Research on Cancer (IARC), which categorizes HPV types 16, 18, 31, 33, 35, 39, 45, 51, 52, 56, 58, and 59 as carcinogenic to humans (Group 1) [23]. The assay employs DPO™ (Dual Priming Oligonucleotide) and TOCE™ (Tagging Oligonucleotide Cleavage and Extension) technologies, providing high analytical sensitivity and specificity. The UDG (Uracil-DNA Glycosylase) system was employed to prevent carryover contamination. Amplification was performed on a CFX96™ Real-Time PCR Detection System (Bio-Rad Laboratories, Hercules, CA, USA). Data analysis and automated result interpretation were carried out using Seegene Viewer software (version 3; Seegene Inc., Seoul, Republic of Korea). In a WHO-organized international HPV genotyping proficiency study, the Anyplex II HPV28 was the sole assay to achieve 100% proficiency across all 11 participating laboratories [24]. The assay has been validated for the detection and genotyping of HPV in anogenital specimens in independent comparative studies [25]. All analyses were performed in accordance with the manufacturer’s instructions at the Institute of Pathology and Molecular Pathology, Helios University Hospital Wuppertal.

### 2.4. Histopathological and Clinical Data Collection

For each specimen, the following histopathological parameters were recorded: tumor entity, specimen type (primary biopsy, recurrence biopsy, re-recurrence biopsy, resection specimen, metastatic specimen), TNM classification, grading (G1–G3), and resection margin status where applicable. Patient-level variables recorded for the histopathological collective included age at initial diagnosis and sex.

### 2.5. Statistical Analysis

Descriptive statistics were used to characterize the study population, with absolute frequencies and percentages for categorical variables, and median with interquartile range (IQR) for continuous variables. Differences between groups were assessed using the Chi-squared test (χ^2^) or Fisher’s exact test for categorical variables, as appropriate. Odds ratios (OR) with 95% confidence intervals were calculated using Woolf’s method, along with Haldane–Anscombe (0.5) correction for zero cells. Type counts between sex groups were compared using the Mann–Whitney U-test. Proportions are reported with 95% Clopper–Pearson exact confidence intervals. Two-tailed *p*-values ≤ 0.05 were considered statistically significant. Given the descriptive nature of this study, *p*-values were not adjusted for multiple comparisons and are interpreted as exploratory. Statistical analyses were performed using Python (version 3.12.3; Python Software Foundation) with the SciPy (version 1.17.1), pandas (version 3.0.2), and NumPy (version 2.4.4) libraries.

## 3. Results

### 3.1. Study Population

A total of 213 tumor specimens from 136 patients (82 female, 60.3%; 54 male, 39.7%) with histologically confirmed squamous cell carcinoma of the anal canal or anal margin were included in the HPV analysis after histopathological review and β-globin quality control. Specimens were obtained from the institutional pathology archive between 2009 and 2020 (retrospective arm 2009–2019; prospective arm from 2020 onwards). Of the 213 specimens, 138 (64.8%), 55 (25.8%), 2 (0.9%), and 18 (8.5%) were pre-therapeutic primary biopsies, recurrence biopsies, re-recurrence biopsies, and specimens from distant metastases, respectively. The median age at initial diagnosis was 62 years (IQR 52–72, range 29–89).

### 3.2. HPV Prevalence Across Specimen Types

HPV DNA was detected in 179 of 213 specimens (84.0%; 95% CI 78.9–88.7%). At the patient level, 125 of 136 patients (91.9%; 95% CI 86.8–96.3%) had at least one HPV-positive specimen. By specimen type, the 34 HPV-negative specimens comprised 13 primary biopsies, 16 recurrence biopsies, and 5 metastatic specimens; the majority derived from otherwise HPV-positive patients, reflecting the decline in HPV positivity across the disease course rather than patient-level HPV-negative disease.

**Figure 2:**
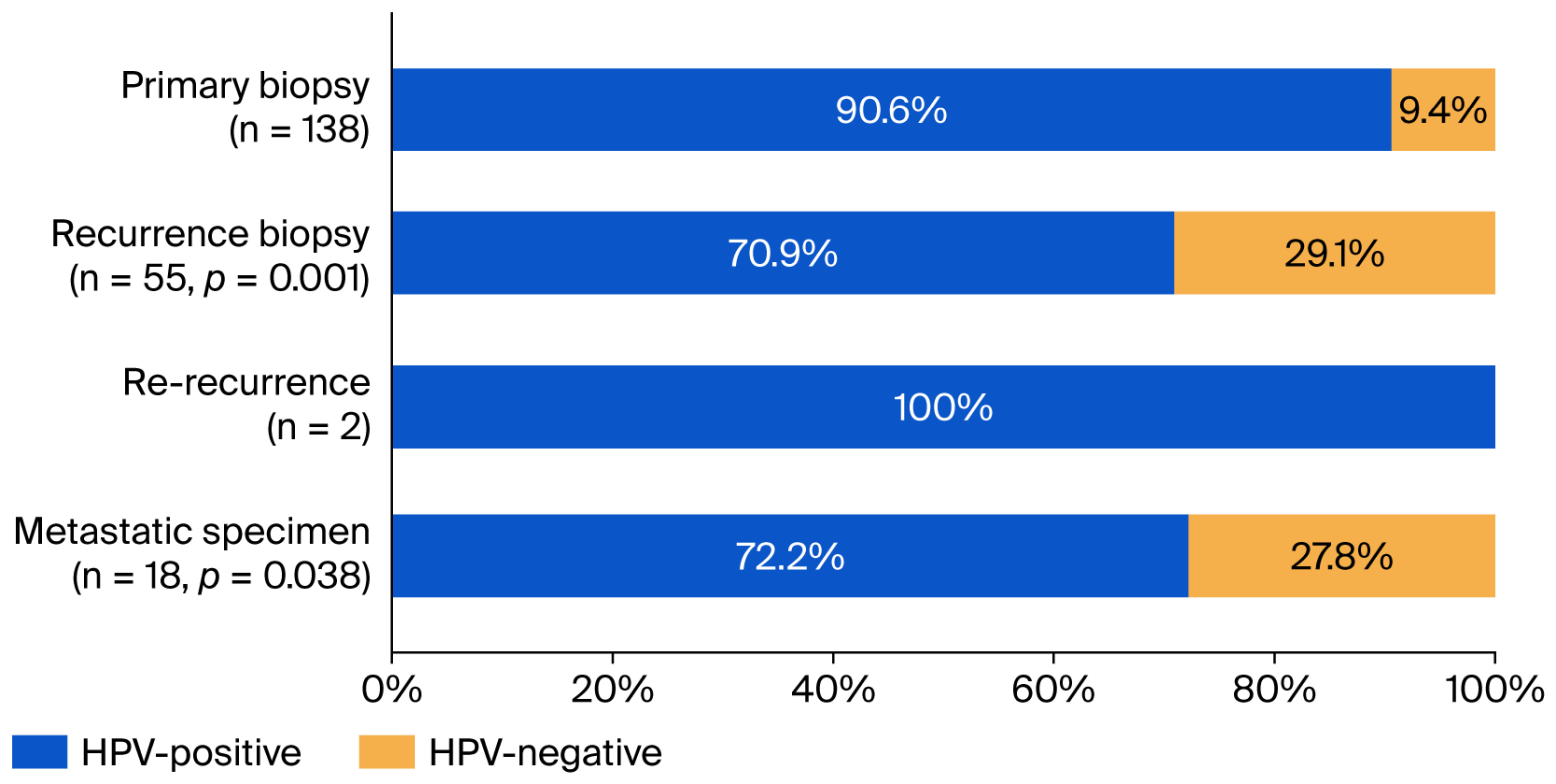
HPV positivity by specimen type. *p*-values are versus primary biopsy (Fisher’s exact test).

HPV positivity rates differed significantly across specimen types (global 2 × 4 Fisher’s exact: *p* = 0.003). Primary biopsies were HPV-positive in 125 of 138 cases (90.6%; 95% CI 84.4–94.9%), recurrence biopsies in 39 of 55 cases (70.9%; 95% CI 57.1–82.4%), and metastatic specimens in 13 of 18 cases (72.2%; 95% CI 46.5–90.3%). Both re-recurrence biopsies were HPV-positive (100%). Compared with primary biopsies, the odds of HPV positivity were significantly lower in recurrence biopsies (OR 0.25; 95% CI 0.11–0.57; *p* = 0.001) and in metastatic specimens (OR 0.27; 95% CI 0.08–0.88; *p* = 0.038). These data are summarized in Figure 2 and Table 1.

### 3.3. HPV Type Landscape

Type distribution was analyzed at two complementary levels: the specimen level, which reflects how often a given type was present in any HPV-positive specimen, and the detection level, which counts each individual type-positive PCR signal separately. Because 24.6% of HPV-positive specimens harbored co-infections (see Section 3.4), a single specimen can yield multiple type detections, and quantifying the relative contribution of each type to the overall genotype landscape requires analysis at the detection level. We distinguish two units of analysis: at the specimen level, each of the 213 specimens is counted once as HPV-positive or HPV-negative, whereas at the detection level, each individual type-specific positive PCR signal is counted separately, so that a co-infected specimen contributes as many detections as genotypes identified within it. Across the 179 HPV-positive specimens, a total of 269 individual type detections were recorded.

Overall, 24 distinct HPV types were identified: 15 high-risk (HR) types (HPV 16, 18, 31, 33, 35, 39, 51, 52, 53, 56, 58, 59, 66, 68, 73) and 9 low-risk (LR) types (HPV 6, 11, 40, 42, 43, 44, 54, 61, 70). Of note, 4 HR types included in the assay (HPV 26, 45, 69, 82) were not detected in any specimen. Of the 269 detections, 219 (81.4%) involved HR types and 50 (18.6%) LR types.

HPV 16 was the dominant type at both analytical levels. At the specimen level, HPV 16 was present in 162 of 179 HPV-positive specimens (90.5%). At the detection level, HPV 16 accounted for 162 of 269 detections (60.2%); the lower detection-level proportion reflects the contribution of additional types detected in co-infected specimens.

**Figure 3:**
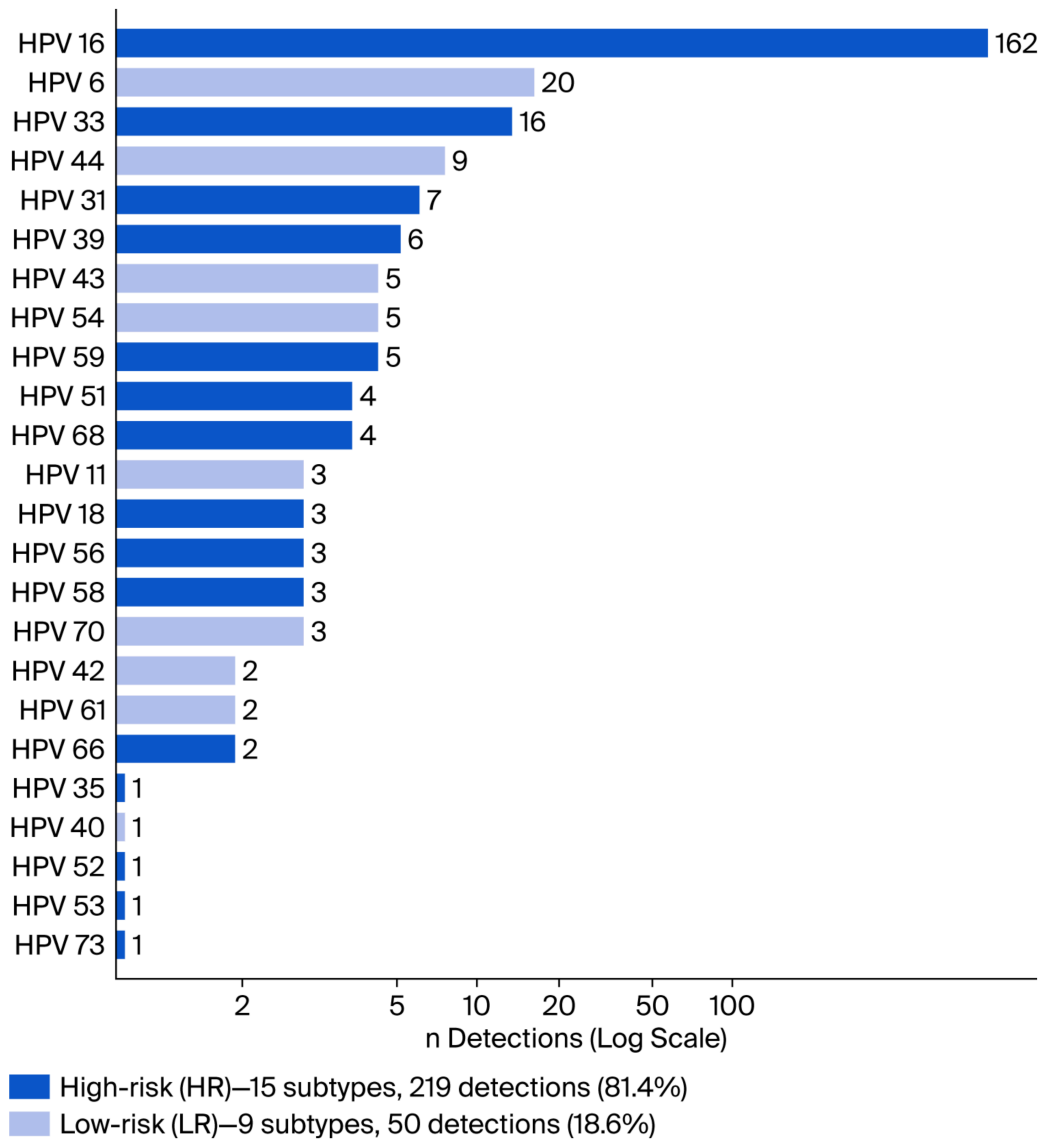
HPV type frequencies across all 269 detections (log scale). HR, high-risk; LR, low-risk.

Beyond HPV 16, 23 types (14 HR, 9 LR) were identified. Non-HPV-16/18 HR types accounted for 54 of 219 HR detections—approximately one in four. The most frequent were HPV 33 (*n* = 16; 5.9%), HPV 31 (*n* = 7; 2.6%), HPV 39 (*n* = 6; 2.2%), HPV 59 (*n* = 5; 1.9%), and HPV 51 and HPV 68 (*n* = 4 each; 1.5%). HPV 18 was detected in 3 specimens (1.1%). In total, non-HPV-16 types contributed 107 of 269 detections (39.8%). Among LR types, HPV 6 was most frequent (*n* = 20; 7.4%), followed by HPV 44 (*n* = 9; 3.3%). Type frequencies across all detections are shown in Figure 3; complete frequencies are listed in Table 2.

### 3.4. Co-Infection Patterns

Of the 179 HPV-positive specimens, 135 (75.4%) harbored a single HPV type, while 44 (24.6%; 95% CI 18.5–31.6%) showed co-infections with two or more simultaneous types. The complete list of co-infection combinations is provided in Table S2. The complexity of co-infections ranged from double infections (*n* = 25; 14.0% of HPV-positive specimens) to an octuple infection involving eight distinct HPV types simultaneously (*n* = 1; 0.6%). Among the 135 single-type infections, HPV 16 was the only type detected in 123 specimens (91.1% of all single-type infections); 132 of 135 single infections were attributable exclusively to HR types, and 3 involved exclusively LR types (HPV 6 in all three cases). The most frequent double-infection combination was HPV 16 and HPV 6 (*n* = 12; 48.0% of double infections). In double and higher-order co-infections, mixed HR and LR combinations predominated; all complex co-infections (quintuple and higher) contained at least one HR type. No LR-only co-infections were observed. Co-infection patterns with HR/LR classification are summarized in Table 3.

### 3.5. HPV Type Spectrum in Recurrence Biopsies

**Figure 4:**
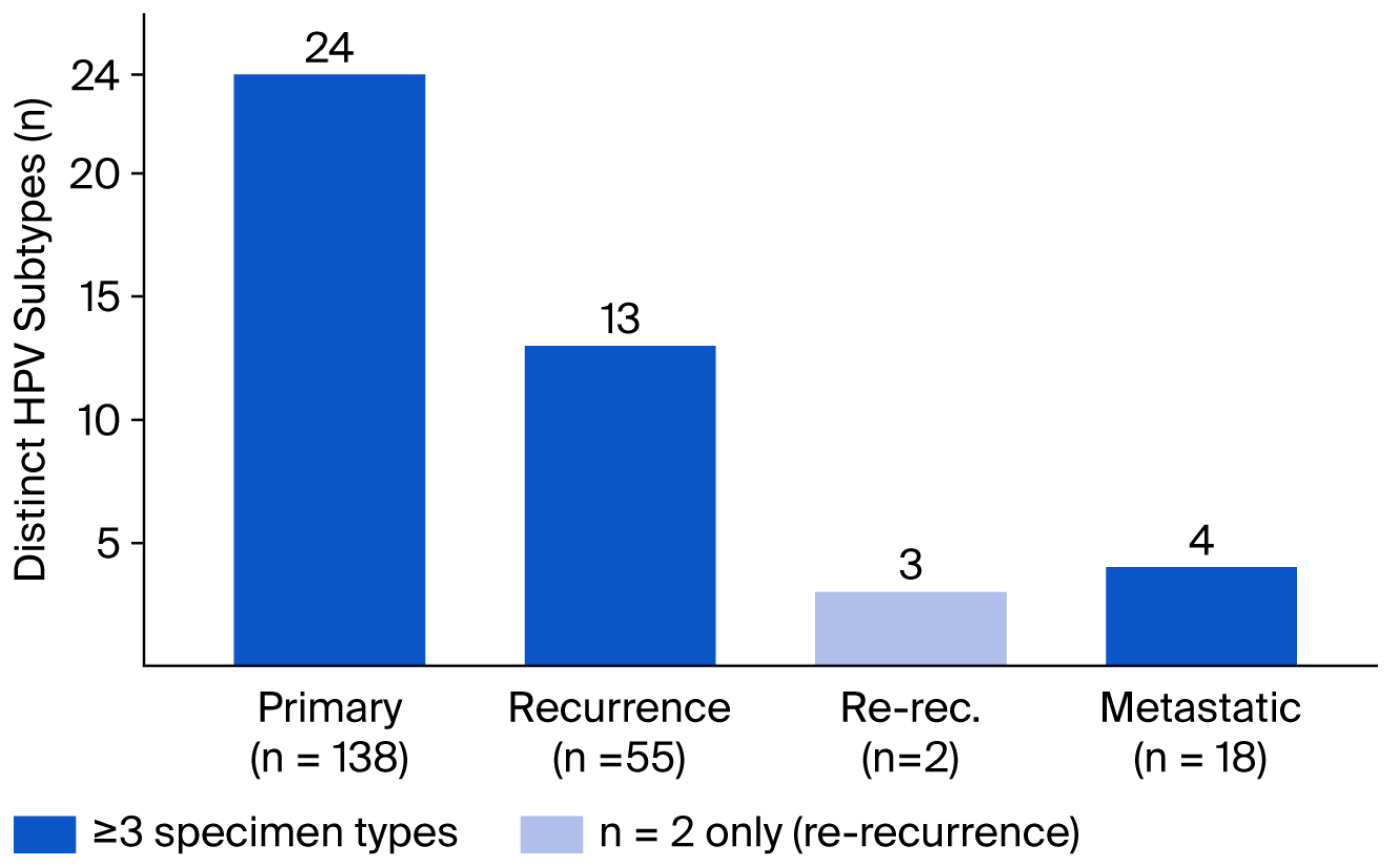
Distinct HPV type diversity by specimen type.

Among the 55 recurrence biopsies, 39 were HPV-positive (70.9%), and 16 were HPV-negative (29.1%). Across these 39 HPV-positive recurrence biopsies, 68 individual HPV detections were recorded, involving 13 distinct types (8 HR, 5 LR; Figure 4). At the specimen level, HPV 16 was present in 36 of 39 HPV-positive recurrence biopsies (92.3%; 95% CI 79.1–98.4%); at the detection level, HPV 16 accounted for 36 of 68 detections (52.9%). Non-HPV-16/18 HR types contributed 18 of 54 HR detections (33.3%). The most frequent non-HPV-16 HR types in recurrence biopsies were HPV 33 (*n* = 5; 7.4% of detections), HPV 31 and HPV 68 (*n* = 3 each; 4.4%), and HPV 51, HPV 59, and HPV 39 (*n* = 2 each; 2.9%). Co-infections were identified in 11 of 39 HPV-positive recurrence biopsies (28.2%; 95% CI 15.0–44.9%), ranging from double to octuple infections.

### 3.6. HPV Type Spectrum in Metastatic Specimens

Among the 18 metastatic specimens from 14 patients, 13 were HPV-positive (72.2%), and 5 were HPV-negative (27.8%). The HPV-positive rate in metastatic specimens was markedly lower than in pre-therapeutic primary biopsies (90.6%). Of note, 4 of the 5 HPV-negative metastatic specimens originated from patients who had received prior chemoradiotherapy, suggesting that treatment-related suppression of HPV DNA to below the assay detection threshold may contribute to HPV-negative results in this setting. The longitudinal dynamics of HPV status across disease progression will be addressed in a forthcoming analysis.

Among the 13 HPV-positive metastatic specimens, high-risk types dominated. HPV 16 was detectable in 11 of 13 cases (84.6%), followed by HPV 33 in 2 cases (15.4%) and HPV 31 in 1 case (7.7%). One HPV-positive metastasis carried HPV 6 as part of a co-infection with HPV 16; no other low-risk types were detected in metastatic specimens. Two HPV-positive metastases harbored HPV 33 as the sole detectable type, without concurrent HPV 16, representing the only HPV-positive metastatic specimens not carrying HPV 16. No metastasis harbored exclusively low-risk HPV types.

**Figure 5:**
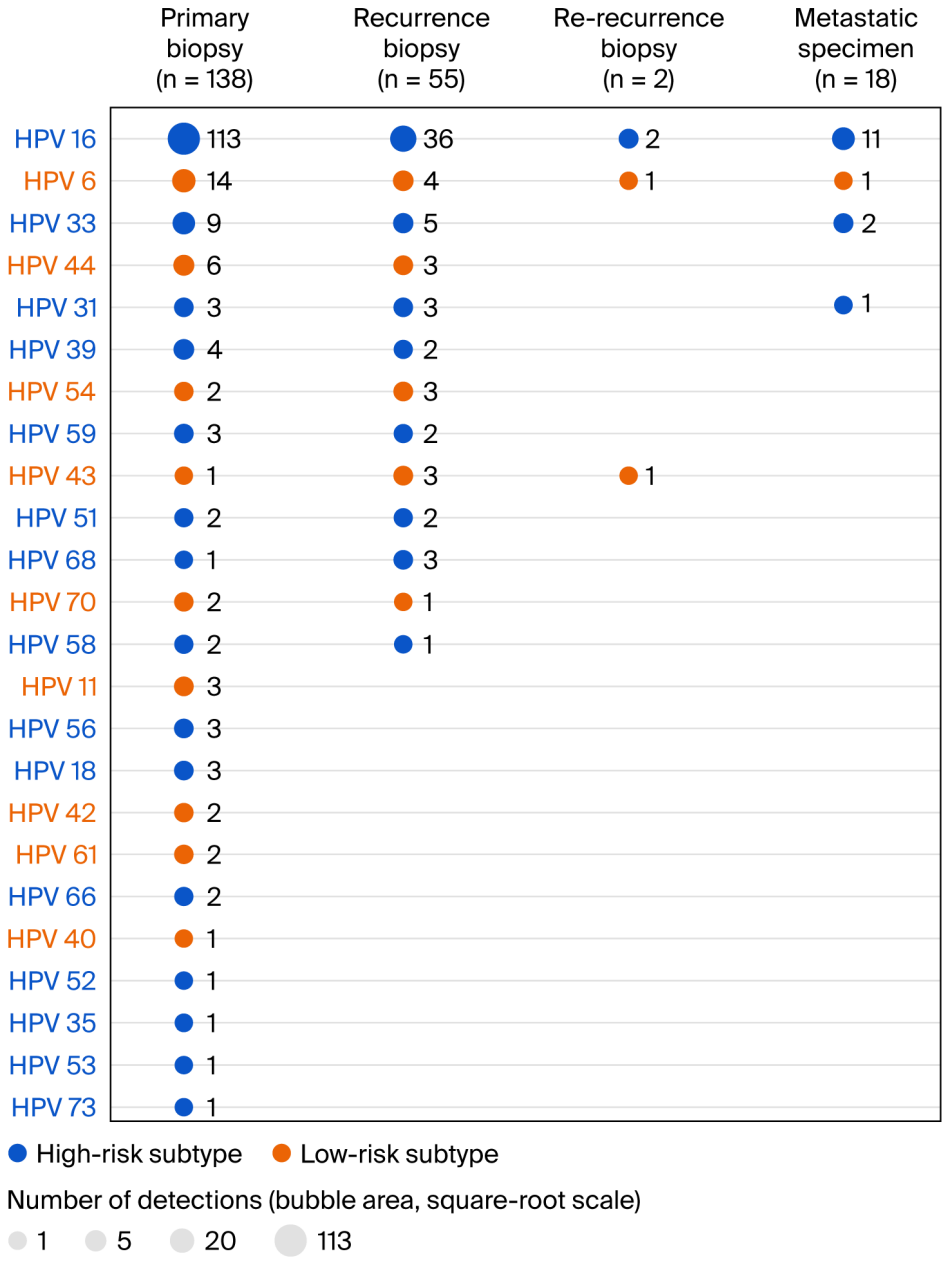
HPV type distribution across specimen types (detection level: 269 detections in 179 HPV-positive specimens). Bubble size reflects the number of detections (square-root scale) with color indicating risk class (high-risk, blue; low-risk, orange). The type spectrum narrows from 24 types in primary biopsies to 4 in metastatic specimens.

Co-infections were identified in 2 of 13 HPV-positive metastatic specimens (15.4%; 95% CI 1.9–45.4%). The detected combinations were HPV 16 + HPV 31 and HPV 16 + HPV 6. In contrast to the broad type diversity observed in primary biopsies, where 24 distinct types were identified, the metastatic type spectrum was substantially narrowed, with only 4 distinct types detected (Figure 5).

### 3.7. Vaccine Coverage: Additional Descriptive Finding

As a supplementary observation, the detected type spectrum was mapped against the coverage of the 9-valent HPV vaccine (Gardasil 9; HPV 6, 11, 16, 18, 31, 33, 45, 52, 58) [21]. Of 269 HPV detections, 215 (79.9%) involved vaccine-covered types. The remaining 54 detections (20.1%) involved types outside Gardasil 9 coverage: 27 of these (10.0% of all detections) were detections of high-risk types not included in the vaccine (HPV 35, 39, 51, 53, 56, 59, 66, 68, 73), and 27 (10.0%) were detections of low-risk types outside vaccine coverage. Two specimens showed carcinogenesis with exclusively low-risk HPV types and no concurrent high-risk detection.

### 3.8. HPV Status and Sex

On the patient level, HPV positivity was assessed for all 136 patients based on the presence of at least one HPV-positive specimen. HPV DNA was detected in 77 of 82 female patients (93.9%; 95% CI 86.3–98.0%) and in 48 of 54 male patients (88.9%; 95% CI 77.4–95.8%). This difference did not reach statistical significance (Fisher’s exact *p* = 0.344), consistent with previously reported trends in HPV-associated anal carcinogenesis [15,17].

Type diversity per patient was comparable between sexes. Single-type infections were observed in 56 of 77 HPV-positive female patients (72.7%) and in 30 of 48 HPV-positive male patients (62.5%). Multi-type infections (≥2 distinct types across all available specimens of a patient) were identified in 21 female patients (27.3%; 95% CI 17.7–38.6%) and in 18 male patients (37.5%; 95% CI 24.0–52.6%; Fisher’s exact *p* = 0.241). The observed range of distinct types per patient was 1–5 in women and 1–12 in men (Mann–Whitney U-test *p* = 0.274). The total spectrum of distinct types was similar in scope, with 20 types across female and 19 across male patients.

## 4. Discussion

This study presents a systematic HPV genotype analysis encompassing pre-therapeutic primary biopsies, recurrences, re-recurrences, and distant metastases in a single-center cohort of 136 SCCA patients with 213 tumor specimens. While larger HPV genotyping cohorts exist in the literature, they have predominantly been restricted to pre-therapeutic primary tumor specimens [12,17,18]. To our knowledge, the present study represents one of the largest analyses to comprehensively genotype HPV across all histopathological specimen types in SCCA, providing a type landscape that extends beyond the primary tumor setting.

### 4.1. Overall HPV Prevalence

HPV DNA was detected in 84.0% and 91.9% of all specimens and patients, respectively, consistent with the reported range of 70–90% in the literature [11,12,26,27]. The lower specimen-level positivity rate compared to the patient-level rate reflects HPV-negative results in recurrent and metastatic specimens from patients with HPV-positive primary tumors, a phenomenon attributable to treatment-related viral suppression or true HPV loss during disease progression. All 13 HPV-negative primary biopsies were positive for the co-amplified β-globin internal control, making a DNA-integrity artifact an unlikely explanation for their negativity; these cases are instead consistent with the well-documented minority of genuinely HPV-independent SCCA, in keeping with our primary-biopsy positivity of 90.6% within the reported literature range.

### 4.2. Type Diversity Beyond HPV 16

While HPV 16 was the dominant type (60.2% of all detections), our data demonstrate a substantially broader HPV landscape than previously appreciated. We identified 24 distinct types, including 15 HR types across 219 individual HR detections. Notably, HPV 18—the second most commonly cited HR type in cervical carcinogenesis—was detected in only 1.1% of our detections, considerably lower than reported for cervical cancer but consistent with other SCCA series [17,18]. This finding confirms that the relative contribution of HPV types to SCCA differs from cervical carcinogenesis, warranting entity-specific profiling rather than extrapolation from cervical cancer data. Of particular clinical relevance, non-HPV-16/18 HR types accounted for 24.7% of all HR detections (54/219), emphasizing that restriction of genotyping to HPV 16/18 alone would miss a substantial proportion of oncogenic viral diversity in this entity.

### 4.3. Co-Infections

Co-infections with two or more HPV types were identified in 24.6% of HPV-positive specimens, with infection complexity ranging up to eight simultaneous types. The predominant pattern was a combination of high- and low-risk types: purely high-risk co-infections were uncommon, and no low-risk-only co-infections were observed. HPV 16 + HPV 6 was the most frequent combination, accounting for 48.0% of all double infections. The biological significance of HPV co-infections in SCCA remains incompletely understood. Whether concurrent low-risk types exert a modulatory effect on the oncogenic activity of high-risk HPV, or represent independent bystander infections without pathogenic relevance, cannot be determined from genotyping data alone and warrants dedicated functional investigation.

### 4.4. HPV in Recurrence Biopsies

Recurrence biopsies showed reduced type diversity compared with the primary tumor setting, with 13 distinct types detected across 39 HPV-positive recurrence specimens. HPV 16 remained the dominant type at both the specimen level (92.3%) and the detection level (52.9%), and non-HPV-16/18 HR types accounted for one third of HR detections (33.3%). Co-infections were observed in 28.2% of HPV-positive recurrence specimens, comparable to the overall co-infection rate of 24.6% across all specimen types. The reduced number of distinct types in the recurrent setting may reflect both the smaller sample size compared with primary biopsies and biological selection during disease progression and prior treatment, although these mechanisms cannot be distinguished within the present descriptive analysis.

### 4.5. HPV in Metastatic Specimens

The type spectrum in metastatic specimens was substantially narrowed compared to the full cohort: only 4 of the 24 distinct types identified across all specimen types were detectable in metastatic tissue. Of particular note, 2 of 13 HPV-positive metastases harbored HPV 33 as the sole detectable type, without concurrent HPV 16. This finding indicates that high-risk types other than HPV 16 were the sole high-risk genotype detected in individual metastatic specimens—a finding not previously documented in systematic genotyping analyses of metastatic specimens. Whether the reduced type diversity in metastatic tissue reflects biological selection, clonal expansion, or the limited power of a small metastatic subcohort warrants systematic investigation in future paired-specimen analyses.

The finding that 4 of 5 HPV-negative metastatic specimens originated from patients who had received prior chemoradiotherapy raises the question of whether treatment-related suppression of viral DNA replication—rather than true oncological HPV loss—contributes to HPV-negative results in this setting. Chemoradiotherapy is known to induce tumor regression and may reduce viral copy numbers below assay detection thresholds without necessarily eliminating integrated HPV sequences from surviving tumor cells [28]. This distinction has potential clinical relevance: an HPV-negative result from a post-treatment metastatic biopsy may not reliably reflect the underlying viral biology of the tumor and should not be equated with primary HPV-negative disease when guiding clinical decisions. Liquid biopsy approaches based on circulating tumor-derived HPV DNA may offer a complementary strategy for HPV status monitoring in this context [29].

### 4.6. Implications for Vaccination and Surveillance

The vaccine coverage data reported here warrant interpretation in their epidemiological context. With a median age at diagnosis of 62 years (IQR 52–72) and a study period covering 2009–2019 and from 2020 onwards, the vast majority of patients in our cohort were born well before the introduction of prophylactic HPV vaccination in Germany (Gardasil approved 2006; STIKO recommendation for ages 9–17). As prophylactic vaccines require administration prior to first HPV exposure to be effective, and as current guidelines do not recommend vaccination in individuals with established infection, our cohort reflects the natural, pre-vaccination HPV type landscape in SCCA. Real-world evidence supports a significant protective effect of HPV vaccination against high-grade anal lesions [30,31], but the impact on anal cancer incidence will only become measurable as vaccinated cohorts reach the age of peak cancer incidence [32].

Given the long latency between initial HPV infection and the development of invasive SCCA—typically estimated at 20–50 years—the impact of current vaccination programs on SCCA incidence will not become measurable for several decades. Cohorts vaccinated from 2006 onwards are unlikely to develop HPV-associated anal cancer before 2040–2060. Against this background, our data provide a pre-vaccination baseline of the type spectrum that will be essential for future surveillance studies designed to quantify the long-term impact of HPV vaccination on SCCA type distribution.

Even under optimistic vaccination scenarios, however, our data indicate that a relevant proportion of SCCA cases would remain attributable to HPV types not covered by the 9-valent vaccine. In our cohort, 20.1% of all HPV detections involved types outside Gardasil 9 coverage: 10.0% were high-risk types not included in the vaccine (HPV 35, 39, 51, 53, 56, 59, 66, 68, 73), and 10.0% were low-risk types outside vaccine coverage. This proportion must be considered alongside the current vaccination reality in Germany: as of 2024, only approximately 50% and 30–34% of 15-year-old girls and boys have received a complete HPV vaccination series, respectively, significantly lower than the WHO target of 90% by 2030 [21]. Even if vaccination coverage were substantially increased, the non-vaccine type burden identified in our study would persist.

These findings support the case for systematic anal cancer surveillance, particularly in high-risk populations, as a complementary strategy to vaccination. The analogy to cervical cancer screening is instructive: routine cytological and HPV-based cervical screening programs have demonstrated that ongoing surveillance remains essential even in partially vaccinated populations, as they detect both vaccine-type and non-vaccine-type disease. For SCCA, no equivalent national screening program currently exists in Germany or most European countries. International consensus guidelines published in 2024 by the International Anal Neoplasia Society now recommend high-resolution anoscopy (HRA)-based screening for HIV-positive men who have sex with men starting at age 35, and for other HIV-positive individuals and solid organ transplant recipients starting at age 45 [33]. Our type data—demonstrating oncologically relevant HPV diversity beyond HPV 16/18 across primary tumors, recurrences, and metastases—provide molecular support for extending surveillance considerations to broader at-risk populations, and underscore that broad-spectrum HPV genotyping should be integral to any future SCCA surveillance program.

### 4.7. Sex Differences

HPV positivity was higher in women (93.9%) than in men (88.9%) on the patient level, though the difference did not reach statistical significance (Fisher’s exact *p* = 0.344). Wessely et al. reported a statistically significant sex difference (90.3% vs. 60.9%, *p* = 0.018) [17]. The attenuated effect in our data may reflect the higher proportion of HIV-positive male patients in our cohort, who tend to approach near-universal HPV positivity rates [15]. Type diversity per patient was comparable between sexes, with similar rates of multi-type infections (27.3% in female versus 37.5% in male HPV-positive patients; *p* = 0.241) and a similar total spectrum of distinct types (20 versus 19).

### 4.8. Clinical Implications

As immune checkpoint inhibitors become integrated into SCCA treatment [8,9], precise knowledge of the tumor-associated HPV type spectrum may inform biomarker development and patient stratification in future immunotherapy trials. Our data—demonstrating 24 distinct types and substantial diversity beyond HPV 16—provide a descriptive foundation for such investigations.

## 5. Limitations

As with any retrospective single-center analysis, the present study has inherent limitations that should be considered when interpreting the findings. Selection bias cannot be excluded, as the cohort reflects the patient population of a single tertiary referral center over a 13-year period. Quantitative viral load data were not systematically recorded, precluding copy number analysis across disease stages. For the metastatic subset, matched primary specimens were available for only a proportion of patients, limiting the interpretability of type comparisons between specimen types. Analyses of HPV status in relation to clinical outcomes are beyond the scope of this descriptive analysis. Certain subgroups were small—particularly the two re-recurrence biopsies and the metastatic subset—so the corresponding estimates carry wide confidence intervals and should be interpreted with caution. In particular, the two re-recurrence biopsies (*n* = 2) are reported descriptively and were not the basis of any inferential comparisons; the pairwise specimen-type comparisons (primary versus recurrence and primary versus metastasis) do not involve this subgroup and are, therefore, unaffected by its inclusion. HPV detection was DNA-based; transcriptional activity was not confirmed by p16 immunohistochemistry or E6/E7 mRNA testing, and DNA recovered from archival FFPE material may be subject to fragmentation and degradation. As HPV detection in archival material depends on specimen type, tumor cellularity, and preprocessing, results derived from such retrospective material must be interpreted with this preanalytical dependency in mind; the co-amplified β-globin control mitigates but does not eliminate this limitation. Notwithstanding these limitations, the size of the cohort, the validated broad-spectrum assay, and the inclusion of all histopathological specimen types provide a robust descriptive foundation for the findings reported here.

Direct comparisons of HPV prevalence and co-infection rates with published series should be interpreted with caution, as observed differences between cohorts may reflect assay sensitivity, type panel breadth, geographic and demographic variation in HPV epidemiology, and patient risk profiles, including HIV status and immunosuppression. These confounders cannot be fully disentangled in a cross-study comparison. The internal consistency of our data—with HPV prevalence figures concordant with established literature ranges and a validated assay with documented 100% proficiency in the WHO international quality assessment [24]—supports the reliability of our findings as a standalone characterization of the HPV genotype landscape in SCCA.

Several limitations warrant mention. First, our descriptive design cannot determine whether the distinct high-risk genotypes identified here differ in biological behavior or therapeutic vulnerability; genotype-specific differences in radiosensitivity, immunogenicity, and response to immune checkpoint blockade have been described in other HPV-associated malignancies, and whether non-HPV-16 high-risk genotypes modify response to chemoradiotherapy, immune checkpoint inhibitors, or emerging HPV-directed therapies in SCCA remains an open question for prospective study. Second, genotyping was performed on bulk tumor tissue and cannot resolve intratumoral heterogeneity; distinct subclones, infiltrating immune-cell populations, and stromal compartments may differ in genotype carriage, and single-cell or spatially resolved approaches will be required to address this. Third, individual HPV vaccination status was not available from the retrospective records; however, with a median age at diagnosis of 62 years over the study period (2009–2019 and from 2020 onwards), the cohort was almost entirely born before the introduction of prophylactic HPV vaccination in Germany (recommended for girls from 2007), so the data reflect the natural, largely pre-vaccination HPV genotype landscape of SCCA.

The detection of low-risk genotypes, particularly HPV 6, within invasive tumor specimens merits comment. Although HPV 6 is classically associated with benign anogenital lesions, it has been demonstrated by laser-capture microdissection within high-grade anal lesions and invasive anal carcinoma [10], suggesting that its presence in a tumor specimen is not necessarily incidental and may reflect a contributory or co-carcinogenic role, particularly in the context of high-risk co-infection. In our cohort, low-risk genotypes were confined to co-infections and were absent from all 34 HPV-negative specimens; notably, the two re-recurrence specimens carrying HPV 6 or HPV 43 alongside HPV 16 were both from HIV-negative patients, indicating that their low-risk carriage cannot be attributed to HIV-related immunosuppression. The etiological agent alone is not always sufficient for disease development, and inter-genotype interactions, chronic inflammation, and immune dysregulation may co-modulate the oncogenic process.

## 6. Conclusions

SCCA harbors a substantially broader HPV type spectrum than previously recognized. Across primary, recurrent, and metastatic specimens, we identified 24 distinct genotypes, with co-infections in 24.6% of HPV-positive specimens. One in four high-risk detections were non-HPV-16/18 types, and high-risk types other than HPV 16 were the sole high-risk genotype detected in individual metastatic specimens. One in five detections comprised types outside 9-valent vaccine coverage. HPV positivity rates were significantly lower across specimen types, while the metastatic type spectrum converged on a small set of high-risk genotypes. Broad-spectrum HPV genotyping beyond types 16 and 18 warrants adoption as standard practice in SCCA diagnostics and surveillance, and potential stratification for HPV-directed therapies. These data provide a descriptive foundation for future studies of HPV-related oncogenesis, treatment response, and outcome.

## Figures and Tables

**Table 1:** HPV prevalence by specimen type.

Specimen Type	*n*	HPV-Positive	HPV-Negative	OR vs. Primary (95% CI)	Fisher’s *p*
Primary biopsy (pre-therapeutic)	138	125 (90.6%)	13 (9.4%)	Reference	—
Recurrence biopsy	55	39 (70.9%)	16 (29.1%)	0.25 (0.11–0.57)	0.001
Re-recurrence biopsy	2	2 (100%)	0 (0%)	n.c.	n.c.
Metastatic specimen	18	13 (72.2%)	5 (27.8%)	0.27 (0.08–0.88)	0.038
Total	213	179 (84.0%)	34 (16.0%)	—	—

**Table 2:** HPV type frequencies (269 total detections in 179 HPV-positive specimens).

Type	Risk Class	*n*	% of All Detections	Gardasil 9 Coverage
HPV 16	HR	162	60.2%	Yes
HPV 6	LR	20	7.4%	Yes
HPV 33	HR	16	5.9%	Yes
HPV 44	LR	9	3.3%	No
HPV 31	HR	7	2.6%	Yes
HPV 39	HR	6	2.2%	No
HPV 43	LR	5	1.9%	No
HPV 54	LR	5	1.9%	No
HPV 59	HR	5	1.9%	No
HPV 51	HR	4	1.5%	No
HPV 68	HR	4	1.5%	No
HPV 11	LR	3	1.1%	Yes
HPV 18	HR	3	1.1%	Yes
HPV 56	HR	3	1.1%	No
HPV 58	HR	3	1.1%	Yes
HPV 70	LR	3	1.1%	No
HPV 42	LR	2	0.7%	No
HPV 61	LR	2	0.7%	No
HPV 66	HR	2	0.7%	No
HPV 35	HR	1	0.4%	No
HPV 40	LR	1	0.4%	No
HPV 52	HR	1	0.4%	Yes
HPV 53	HR	1	0.4%	No
HPV 73	HR	1	0.4%	No

**Table 3:** Co-infection patterns in HPV-positive specimens. HR = high-risk type; LR = low-risk type.

Infection Pattern	*n* (Specimens)	% of HPV-Positive	HR Only	HR + LR	LR Only
Single	135	75.4%	132	0	3
Double	25	14.0%	11	14	0
Triple	9	5.0%	3	6	0
Quadruple	1	0.6%	0	1	0
Quintuple	5	2.8%	1	4	0
Sextuple	1	0.6%	0	1	0
Septuple	2	1.1%	0	2	0
Octuple	1	0.6%	0	1	0

## Data Availability

The data presented in this study are available upon request from the corresponding author due to patient data protection regulations.

## Supplementary Materials

The following supporting information can be downloaded at: https://www.mdpi.com/article/10.3390/cancers18152396/s1, Table S1: STROBE Checklist—Reporting of Observational (Cross-Sectional/Cohort) Studies; Table S2: Complete list of HPV co-infection combinations identified across all 213 specimens.
