# Supplementary material for: HPV Genotype Landscape in Anal Squamous Cell Carcinoma Across Primary, Recurrent, and Metastatic Tumor Specimens
Source: Cancers (Basel). 2026 Jul 25;18(15):2396. doi: 10.3390/cancers18152396 (PMC13465161; doi:10.3390/cancers18152396)
Supplement: Supplementary file 1 [file cancers-18-02396-s001.zip › cancers-4411491-supplementary.pdf]

Table S1. STROBE Checklist — Reporting of Observational (Cross-Sectional/Cohort) Studies.

Section / Topic	Item	Recommendation	Reported in (section; page)
Title and abstract	1	(a) Study design indicated in the title/abstract; (b) balanced summary of design, methods, results.	Title; Abstract, p. 1
Introduction — Background/rationale	2	Scientific background and rationale.	Introduction, pp. 1–3
Introduction — Objectives	3	Specific objectives / aims.	Introduction (final paragraph), p. 3; Abstract, p. 1
Methods — Study design	4	Key elements of study design stated early.	Methods 2.1, pp. 3–5
Methods — Setting	5	Setting, locations, relevant dates (recruitment, exposure, data collection).	Methods 2.1, 2.2 (2009–2019 and from 2020 onwards), pp. 3–5
Methods — Participants	6	Eligibility criteria, sources, and methods of selection.	Methods 2.2, pp. 3–5; Figure 1, p. 4
Methods — Variables	7	Outcomes, exposures, predictors, confounders, effect modifiers defined.	Methods 2.4, pp. 3–5
Methods — Data sources/measurement	8	Sources of data and details of assessment methods (HPV genotyping assay).	Methods 2.3, 2.4, pp. 3–5
Methods — Bias	9	Efforts to address potential sources of bias.	Methods 2.2, pp. 3–5; Limitations (Section 5), pp. 15–16
Methods — Study size	10	How the study size was arrived at.	Methods 2.2, pp. 3–5; Results 3.1, pp. 5–6; Figure 1, p. 4
Methods — Quantitative variables	11	Handling of quantitative variables / groupings.	Methods 2.5, pp. 3–5
Methods — Statistical methods	12	All statistical methods, subgroup/sensitivity analyses, handling of missing data.	Methods 2.5, pp. 3–5
Results — Participants	13	Numbers at each stage; reasons for exclusion; flow diagram.	Results 3.1, pp. 5–6; Figure 1, p. 4
Results — Descriptive data	14	Characteristics of study participants and specimens.	Results 3.1, pp. 5–6
Results — Outcome data	15	Numbers of outcome events / summary measures (HPV positivity).	Results 3.2, pp. 6–11; Table 1, p. 6
Results — Main results	16	Estimates and precision (95% CI); category boundaries.	Results 3.2–3.8, pp. 6–11; Table 1, p. 6; Table 2, p. 8; Table 3, p. 9
Results — Other analyses	17	Other analyses (subgroups: vaccine coverage, sex).	Results 3.7, 3.8, pp. 6–11
Discussion — Key results	18	Key results with reference to objectives.	Discussion (opening; Section 4.1), p. 12
Discussion — Limitations	19	Limitations; sources of bias/imprecision; direction/magnitude.	Limitations (Section 5), pp. 15–16

Section / Topic	Item	Recommendation	Reported in (section; page)
Discussion — Interpretation	20	Cautious overall interpretation considering objectives and evidence.	Discussion (Section 4), pp. 12–15
Discussion — Generalisability	21	External validity / generalisability.	Limitations (Section 5), pp. 15–16; Clinical Implications (Section 4.8), pp. 12–15
Other information — Funding	22	Funding source and role of funders.	Funding statement, p. 16

HPV Genotype Landscape in Anal Squamous Cell Carcinoma Across Primary, Recurrent, and Metastatic Tumor Specimens. Page numbers refer to the final proof version of the manuscript.

Table S2. Complete list of HPV co-infection combinations identified across all 213 specimens.

Combination	Complexity	HR/LR Classification	*n*
HPV 16 + HPV 6	Double	HR + LR	12
HPV 16 + HPV 31	Double	HR only	3
HPV 16 + HPV 33	Double	HR only	2
HPV 16 + HPV 68	Double	HR only	2
HPV 16 + HPV 11	Double	HR + LR	1
HPV 16 + HPV 18	Double	HR only	1
HPV 16 + HPV 39	Double	HR only	1
HPV 16 + HPV 44	Double	HR + LR	1
HPV 16 + HPV 58	Double	HR only	1
HPV 16 + HPV 59	Double	HR only	1
HPV 16 + HPV 58 + HPV 6	Triple	HR + LR	2
HPV 16 + HPV 43 + HPV 6	Triple	HR + LR	1
HPV 16 + HPV 52 + HPV 6	Triple	HR + LR	1
HPV 16 + HPV 33 + HPV 18	Triple	HR only	1
HPV 16 + HPV 68 + HPV 31	Triple	HR only	1
HPV 16 + HPV 70 + HPV 44	Triple	HR + LR	1
HPV 16 + HPV 51 + HPV 68	Triple	HR only	1
HPV 51 + HPV 33 + HPV 42	Triple	HR + LR	1
HPV 16 + HPV 6 + HPV 51 + HPV 54	Quadruple	HR + LR	1
HPV 16 + HPV 35 + HPV 53 + HPV 56 + HPV 73	Quintuple	HR only	1
HPV 33 + HPV 39 + HPV 31 + HPV 43 + HPV 44	Quintuple	HR + LR	1
HPV 66 + HPV 16 + HPV 56 + HPV 54 + HPV 44	Quintuple	HR + LR	1
HPV 66 + HPV 16 + HPV 70 + HPV 44 + HPV 11	Quintuple	HR + LR	1
HPV 59 + HPV 16 + HPV 56 + HPV 42 + HPV 11	Quintuple	HR + LR	1
HPV 51 + HPV 33 + HPV 39 + HPV 43 + HPV 61 + HPV 44	Sextuple	HR + LR	1
HPV 59 + HPV 16 + HPV 33 + HPV 31 + HPV 43 + HPV 54 + HPV 44	Septuple	HR + LR	1

HPV 59 + HPV 33 + HPV 39 + HPV 54 + HPV 61 + HPV 44 + HPV 40	Septuple	HR + LR	1
HPV 59 + HPV 33 + HPV 39 + HPV 31 + HPV 43 + HPV 54 + HPV 70 + HPV 44	Octuple	HR + LR	1

Table S2 lists all co-infection combinations detected in 44 of 179 HPV-positive specimens (24.6%). Single infections (*n* = 135) and specimens with no detectable HPV (*n* = 34) are not shown. HR = high-risk HPV type; LR = low-risk HPV type. Types are listed in the order of their detection frequency within the assay output. Abbreviations: HR, high-risk; LR, low-risk; *n*, number of specimens. HPV types classified as high-risk (HR): 16, 18, 26, 31, 33, 35, 39, 45, 51, 52, 53, 56, 58, 59, 66, 68, 69, 73, 82. HPV types classified as low-risk (LR): 6, 11, 40, 42, 43, 44, 54, 61, 70.
